# A New Purpose to Old Arsenal: Exploring the Safety and Efficacy of a New Regimen Comprising a Combination of Chlorhexidine Gluconate and Cetrimide for Seborrheic Dermatitis Treatment in a Randomized Placebo-Controlled Clinical Trial

**DOI:** 10.7759/cureus.70138

**Published:** 2024-09-25

**Authors:** Rashmi Mehta, Diamond Jain, Pratheesh N Prabhakaran, Deva Kumari, Vijayan Padmanabhan, Kulpreet Bhui, Deepa Murali

**Affiliations:** 1 Savlon Swasth India Mission, ITC Life Sciences and Technology Centre, Bangalore, IND

**Keywords:** antiseptic, cetrimide, chlorhexidine, dandruff, malassezia, scalp, zinc pyrithione

## Abstract

Background: Seborrheic dermatitis is a common scalp condition affecting the quality of life of individuals across all age groups. The uninhibited proliferation of *Malassezia spp.* and enhanced sebaceous gland activity often leads to scalp flaking, mild erythema, and itching, thereby worsening the situation. Here, we aimed to study the efficacy of an antiseptic liquid as a pre-shampoo rinse followed by a non-anti-dandruff shampoo as a dandruff care strategy.

Methods: The anti-dandruff efficacy of a chlorhexidine-cetrimide-based antiseptic liquid (ASL) as a pre-shampoo scalp rinse, followed by a regular non-anti-dandruff shampoo, was compared with a commercial Zinc pyrithione (ZnPTO) based anti-dandruff shampoo following a half-head paired treatment design in a randomized, placebo-controlled, double-blind trial. The study was conducted on 50 healthy human adults of both genders with moderate to severe dandruff. During the 12 weeks of this study, the product's safety and efficacy were evaluated based on the dermatologist's visual assessment, the subjects' self-assessment, the loose flake density score, and hair fall.

Results: ASL as a pre-shampoo scalp rinse demonstrated significant reduction (p<0.001 for n=47) in dandruff and itching after the treatment and the regression phase, performing at par with a marketed anti-dandruff shampoo. Moreover, ASL was established to be safe, non-irritant, and well-tolerated. No product-related adverse event, discomfort, or irritation symptoms were recorded, having any impact on hair sensory properties.

Conclusions: This study demonstrates that a regimen of a chlorhexidine+cetrimide antiseptic liquid for scalp treatment followed by rinse with a regular shampoo can prove as effective as a ZnPTO-based anti-dandruff shampoo in reducing dandruff symptoms for individuals with moderate dandruff.

## Introduction

Dandruff, also known as seborrheic dermatitis, is a common scalp condition characterized by excessive scaling or flaking of the skin, scalp dryness or oiliness, redness, and itchiness of the scalp [1]. Three prime etiologies are associated with seborrheic dermatitis, which includes the presence of commensal yeast of *Malassezia spp.*, excessive secretions of the sebaceous glands, and the individual’s sensitivity [2]. *Malassezia spp.* is the normal skin commensal, lipophilic yeasts widely attributed to skin disorders like seborrheic dermatitis, which together affect more than 50% of human beings [3]. 

Various species of *Malassezia* are present on the scalp, and they thrive by utilizing the scalp sebum. *M. restricta* and *M. globosa* are present in varying proportions in the scalp microbiota of the population of different origins. These yeast species secrete lipases and hydrolases that break down human sebum and release diglycerides and unsaturated and saturated fatty acids on the scalp [4]. Though most of the saturated long-chain fatty acids (LCFA) such as palmitic acid are consumed by the fungus itself for its own growth, some of these unsaturated fatty acids penetrate the stratum corneum, induce inflammation, and enhance abnormal keratinization in individuals with sensitive and compromised skin [5]. During this inflammatory process, multitudes of soluble factors participate, and some of them alter the normal functioning of the epidermis. This results in hyperproliferation and rapid transfer of incompletely keratinized cells to the surface, which then appear as clumps or flakes, known as dandruff [6].

The well-known antiseptic combination of chlorhexidine and cetrimide has been efficient in reducing the load of *Malassezia spp.* in *in vitro* settings. Similarly, our initial *in vitro* studies exhibited significant anti-fungal effects of an antiseptic liquid (ASL) formulation containing chlorhexidine and cetrimide against various *Malassezia spp.* This was followed by a clinical trial to assess the safety and efficacy of this antiseptic liquid as a scalp pre-rinse followed by a non-anti-dandruff shampoo wash. This study was a single-centered, double-blinded, controlled, randomized, comparative, and parallel design study. A scalp pre-rinse of placebo antiseptic liquid followed by a wash with conventional anti-dandruff shampoo containing zinc pyrithione (ZnPTO) was included for comparison.

## Materials and methods

Preparation and in vitro assessment of anti-fungal activity of antiseptic liquid

The antiseptic liquid was manufactured in Savlon Swasth India Mission Laboratory, ITC Limited, Bangalore, India. This antiseptic liquid comprises chlorhexidine gluconate solution IP 1.5% v/v and cetrimide IP 3.0% w/v, both of which are well-established antimicrobial agents [7,8]. The anti-fungal activity of the antiseptic liquid was determined as per the standard guidelines mentioned in IS-11479-2 (2001) at a final dilution of 1:15 (6.25% of the initial concentration).

Clinical study design

A single-centered, randomized (1:1), double-blind (investigator and subjects), parallel-group, placebo-controlled clinical trial following a half-head application design was conducted at M/s MASCOT SPINCONTROL, a Clinical Research Organization based in Mumbai, India. This study was carried out in compliance with the E6 Note for Guidance on Good Clinical Practices (CPMP/ ICH/ 135/ 95/ 5) [9] and the principles of the Declaration of Helsinki [10], Indian Council of Medical Research (ICMR) guidelines, and in compliance with the Indian government’s regulations, guidelines, and standards applicable to such clinical studies [11]. An independent ethics committee, the “Suraksa Ethics Committee," reviewed and approved the study protocol. The study was prospectively registered on the Clinical Trial Registry of India (CTRI) with the registration number CTRI/2017/05/008658 (Registration Date: 25/05/2017).

**Figure 1:**
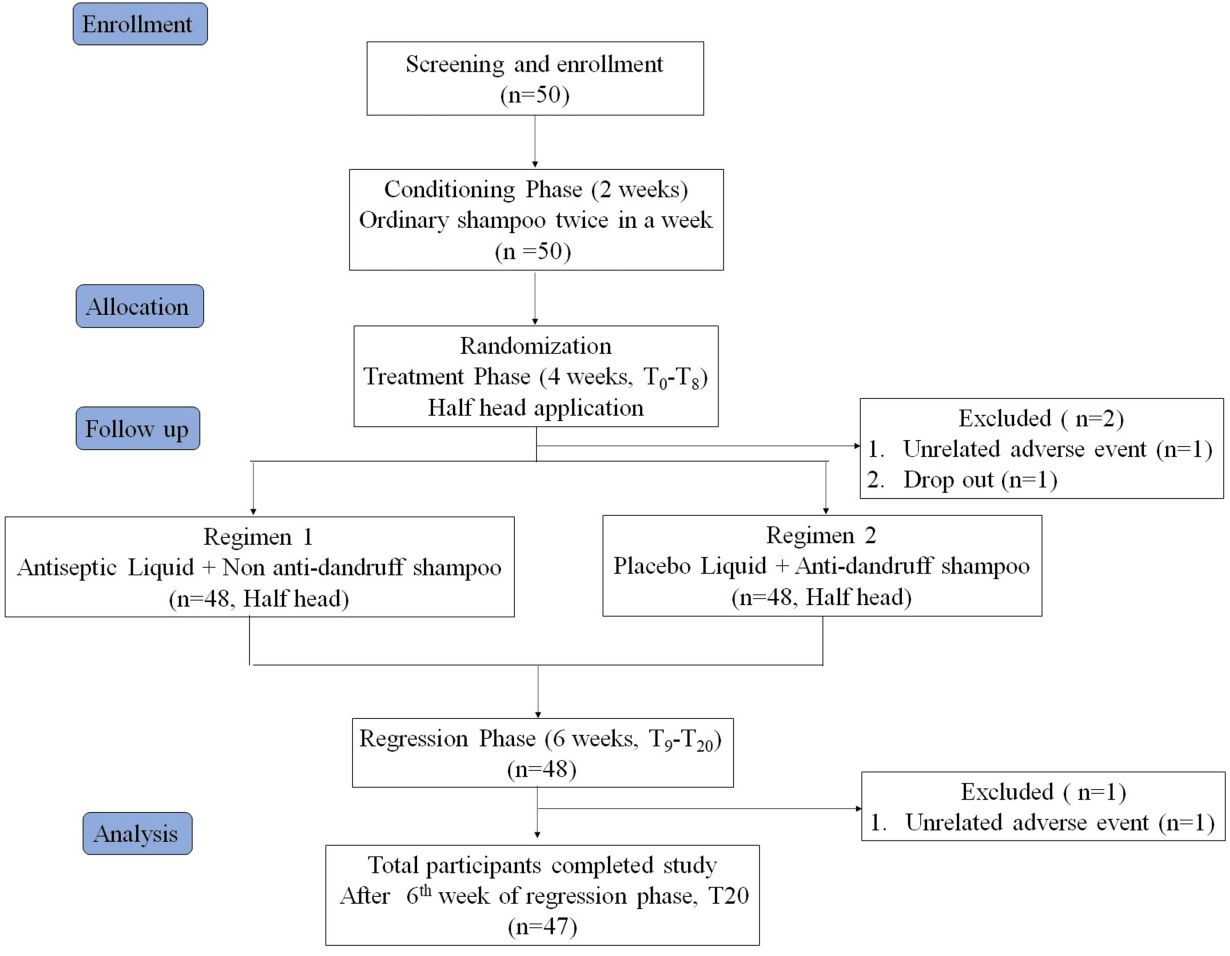
Flow diagram showing the clinical study design

The study comprised two treatment regimens; regimen one included antiseptic liquid followed by the use of non-anti-dandruff shampoo, and regimen two included placebo antiseptic liquid followed by the use of zinc pyrithione (ZnPTO) containing commercially available anti-dandruff shampoo. The duration of the study was 12 weeks, including two weeks of the conditioning phase, four weeks of the treatment phase, and six weeks of the regression phase (Figure 1).

Inclusion criteria 

The subjects were selected based on their willingness to participate for the whole study duration, and a preliminary interview was conducted to explain the study details, like risks and benefits, and thereafter the informed consent form was signed by the subjects. Fifty (50) Indian adult men and women, aged between 20 and 45 years, with moderate to severe dandruff and pruritus on both sides of the scalp and sufficient length of hair to allow parting into a clear section for scalp examination, were selected. All the participants were well tolerant to the ingredients of the investigational products based on a skin sensitivity test, and none of the subjects had participated in a similar investigation in the past four weeks. These participants had no infectious or evolutive pathology, which could make them vulnerable and stop the study midway.

The individuals were excluded from the study if they had excessive fair or white hair or very short hair; dermatosis on the scalp; a history of surgery on the scalp; displayed folliculitis or tinea capitis; regular users of anti-dandruff shampoo; or severe hair fall due to endocrine disorder. Other reasons included, they were pregnant or nursing; having a history or existing condition of an allergic response to any hair care products; suffering from any communicable skin/scalp infection; other skin manifestations like psoriasis, dermatitis, or other dermatoses, which could possibly interfere with the assessments or study results; had severe hair fall due to endocrine disorders; individuals undergoing chronic or intermittent medical treatments, including medications like steroids, antihistamines, antimicrobials at the time of screening or in the past 3-6 months, which would compromise the study results, or were ailing from systemic and/or chronic illness or were under therapy for dandruff. Written informed consents were obtained from each participant before their enrolment in the study. Three participants left the study during the investigation, none related to product-induced adverse events.

Blinding or masking

Study participants were allocated an identifier number based on the order of their enrolment. Participants received both treatment regimens on the designated sides of their scalp (right and left) as per the pre-specified randomization schema. The products were concealed by using codes that both the participants and investigators were unaware of, thereby ensuring that the study was double-blind.

Restrictions

Subjects were abstained from using any hair care products (leave on and rinse off). Only the mild non-anti-dandruff shampoo was used by the subjects during the conditioning phase as per the study protocol. During the treatment and regression phase, the subjects were restricted from using any kind of hair care products, including the provided mild non-anti-dandruff shampoo, and washing or wetting their hair at home was also prohibited. The subjects were also prohibited from using any medication, including aspirin-based products, anti-inflammatory drugs, steroids, anti-coagulants, antimicrobials like antifungals and antibiotics, cortico therapy, anti-histamines, etc., by the general or local route.

The procedure adopted for a clinical study

Conditioning Phase

Subjects washed their scalp with a non-anti-dandruff shampoo twice a week for two weeks at home, amounting to a total of four washes. Post-conditioning, subjects visited the site as per the specified schedule for the treatment phase.

Treatment Phase

Subjects were acclimatized to the ambient conditions (RT: 20ºC to 25ºC, RH 50% ±10%) of the study center for nearly 30 minutes. Their hair was partitioned into eight sections to aid assessment (Appendix [Figure 5]), and baseline assessment was carried out. Further, the subjects’ hair was combed and partitioned into two vertical half sections (Appendix [Figure 5]). Both the left and right sections of the hair were combed using an equal number of strokes. Subjects underwent the first treatment with the designated antiseptic liquid (Test ASL or Placebo ASL, both diluted 1:15 times in distilled water) on the specified side of the scalp, as per the randomization plan. The solution was applied in small volumes spread across two to three times to ensure complete saturation of the scalp with the product and uniformity in dosage between products and across subjects. Adequate time was given for the previously loaded liquid to spread well before loading the subsequent volume to prevent product wastage due to dripping. The antiseptic liquid was retained on the scalp for at least 10-15 minutes, followed by washing using the shampoo. Both sides of the scalp were thoroughly rinsed with 4.0 mL of the designated shampoo (Mild Non-AD Shampoo or AD Shampoo). Adequate care was taken to avoid cross-contamination of the products on either side of the scalp. Each section of the hair was towel-dried, combed, and re-sectioned for post-treatment evaluation. Subjects visited the study center twice a week with at least a two-to-three-day gap between the consecutive visits and completed about eight sessions of controlled treatment with the investigational products on the designated sites.

Regression Phase

During the regression phase, subjects underwent 12 sessions of controlled washes with a non-anti-dandruff shampoo over six weeks. The assessments were carried out at different time points (as mentioned in Appendix [Table 3]).

Efficacy assessment of the test products

The evaluation was carried out based on the subject’s self-evaluation, dermatological evaluation for efficacy, assessment of the area covered by loose flakes, and counting of fallen hair after standardized combing on the eight sections of the scalp. Prior to evaluation, both the left and right sections of the hair were combed using an equal number of strokes. Dermatologists assessed the overall product efficacy based on the reduction in dandruff severity (Appendix [Table 4]). The scores were obtained following a scoring system adapted and modified from the Adherent Scalp Flaking Score (ASFS) [12]. The modified scoring system involved the calculation of the product of the score for the affected area and the dandruff severity for each section, and finally the addition of the products of all four sections on a specific side of the scalp. This led to the possibility of a total dandruff score ranging from 0 to 100 for each half scalp, with 0 representing a normal scalp, free of dandruff, and 100 representing the most severe dandruff. The scalp pruritus severity was evaluated on a 5-point scale (Appendix [Table 5]). The area covered by loose dandruff flakes and the hair fall was assessed visually by collecting the fallen flakes and hairs while combing each half side of the scalp on separate black papers (black card method). The area covered by loose dandruff flakes was scored on a scale of 1 to 6 (Appendix [Table 6]). The subject’s self-evaluation was based on the questionnaire, where they scored the scalp and hair condition (Appendix [Table 7]).

Assessment of the safety of products

Dermatologists assessed the safety of the product through the grading on each half side for the defined clinical symptoms and functional sensations of the participants. The clinical symptoms included erythema, edema, dryness, scaling, and peeling, while the functional sensations included itching, tingling, stinging, and burning. The severity of the symptoms was evaluated using a scale ranging from none, mild, moderate, severe, to very severe (Appendix [Table 8]).

Statistical analysis methods

Anticipating a high dropout rate (43%) considering the long duration and multiple visits of the study, we enrolled 50 (n=50) subjects in the study. Statistical analysis was conducted using the “Compare Groups package in R” to determine the significant difference in dandruff symptoms post-treatment over baseline and between the treatments. A paired t-test was used to analyze continuous data, while the Chi-Square/Fisher Exact test was applied to analyze categorical data. The missing, unused, and spurious data were excluded from the analysis. A p-value of < 0.05 was considered significant.

## Results

Antiseptic liquid exhibited in vitro anti-fungicidal activity against *Malassezia spp.*

Based on the previous findings, we conducted *in vitro* assays to determine the efficacy of ASL against various *Malassezia spp.* ASL exhibited >4 log_10_ reduction in the viability of the tested *Malassezia spp.* within 1 minute of contact (Table 1). Therefore, clinical studies were carried out with 50 subjects having moderate to severe dandruff enrolled in the study.

**Table 1:** Antimicrobial efficacy of antiseptic liquid based on one minute contact kill assay

Organism name	Log reduction (CFU/mL)	Percentage Reductions
Malassezia globosa NBRC 101597	4.92	>99.99
Malassezia pachydermatis MTCC 1369	4.61	>99.99
Malassezia furfur ATCC 14521	4.58	>99.99
Malassezia restricta ATCC MYA 4611	4.94	>99.99

Adverse events or withdrawal from the study

Out of 50 participants enrolled in this study, two adverse events occurred unrelated to the investigational product, while one was discontinued owing to personal reasons. Therefore, 47 (n=47) subjects were considered in the expression of results and final evaluation of the investigation, which included 24 females (~51%) and 23 males (~49%). The mean age of the study participants was ~28.6 years.

Dermatologists' evaluation of the product efficacy

**Figure 2:**
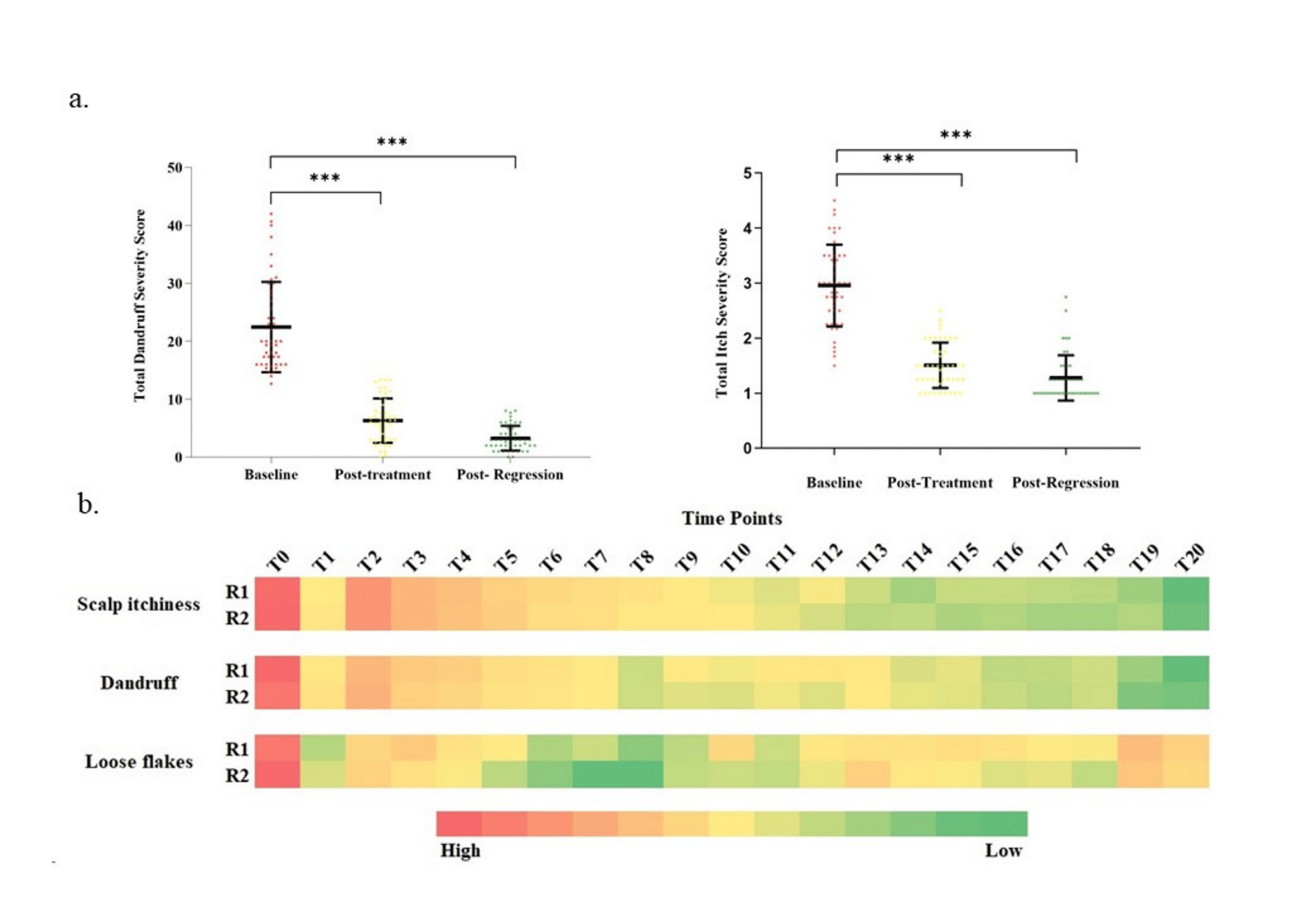
Dermatologist’s assessment of dandruff severity and scalp pruritis a. Total dandruff and itch severity scores comparison between the baseline and the post-treatment and post-regression phase of ASL as scalp pre-rinse (Regimen 1), ***p-value<0.001 at each studied timepoint vs. baseline, by paired t test b. Heat map depiction of the comparison of Regimen 1 and Regimen 2 for all the study time-points, p-value >0.05, by independent t test.

Based on the dermatologist's assessment, it was observed that the study population had moderate dandruff severity at baseline. The total dandruff severity for regimen 1 significantly decreased after the treatment phase and the regression phase (p<0.05), as depicted in Figure 2a. Overall, a mean reduction of ~71.89% and ~85.47% was observed after the treatment phase and the regression phase, respectively. Similarly, a statistically significant reduction in Dandruff with regimen 2 was observed at all studied time points compared to the baseline (p<0.001). Both regimens exhibited similar reductions in total dandruff severity score, showing no significant difference at any of the studied time points, indicating parity performance in both speed and extent of response (Figure 2b). 

A statistically significant reduction in scalp itchiness was observed at all studied time points compared to the baseline, starting from the 1st application for both regimens (p<0.001). Moreover, no difference was observed in the scalp pruritus for the subjects undergoing different treatments with different regimens. (Figure 2b). The study population had a similar extent of loose dandruff flakes at baseline. Both regimens conferred a significant reduction in loose dandruff flakes over baseline at all the studied time points. No significant difference in loose flakes was observed between the regimens at any of the studied time points (Figure 2b). Therefore, based on these results, it can be interpreted that the use of antiseptic as a pre-rinse helps in the reduction of dandruff severity and scalp itchiness as assessed by a dermatologist.

Investigators' assessment of hair fall

**Figure 3:**
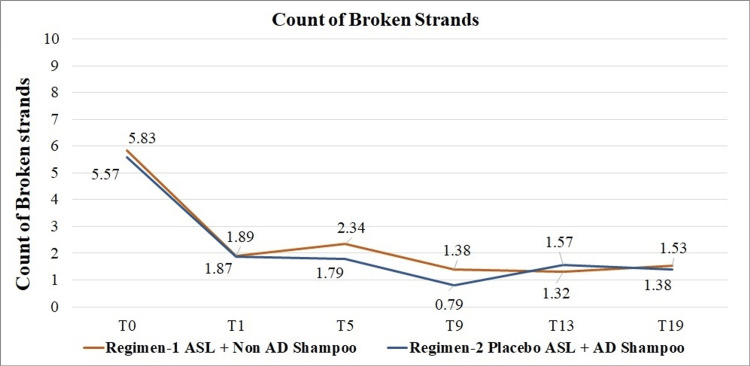
Hair fall assessment with respect to count of broken strands, p-value >0.05, by paired t-test for comparison of change over baseline

Increased dandruff is often associated with hair fall. A statistically significant reduction in hair fall due to strand breakage was seen for both Regimen 1 and Regimen 2 at all studied time points over baseline (p<0.05). No significant difference was observed between the studied regimens in the count of full-length strands or broken strands at any of the studied time points (Figure 3). Further, we assessed the safety of both regimens based on the clinical symptoms and functional sensations.

Dermatologists' assessment of product safety

The product safety was measured based on the clinical and functional sensations occurring during the study. As discussed previously, the clinical symptoms considered for safety assessment included erythema, edema, dryness, scaling, peeling, itching, tingling, stinging, and burning. Neither of the regimens caused any adverse event, irritation, or discomfort. None of the subjects showed the symptoms of erythema, edema, tingling, peeling, stinging, and burning during and at the end of the study for the regimen using ASL as a pre-rinse scalp. Both regimens conferred a significant reduction in scalp dryness over baseline at all studied time points (p<0.05). The first regimen exhibited reduced scaling (p<0.05) at all the time points compared to baseline, while the second regimen showed reduced scaling at a significance level of p<0.05 at all the studied time points except T2 and T4 (p<0.1). Moreover, symptoms like dryness, scaling, and itchiness were significantly (p<0.001) improved after the treatment and the regression phase (Table 2). There were two instances of non-product-related mild adverse events that resolved within two days of being reported. However, product application was discontinued for these two subjects.

**Table 2:** Dermatologist assessment of product safety, *p-value basis paired t-test Regimen 1: Antiseptic liquid followed by non-anti-dandruff shampoo, Regimen 2: Placebo liquid followed by anti-dandruff shampoo.

	Regimen 1					Regimen 2				
Symptoms [n (%)]	Baseline (n=47)	Post Treatment (n=47)	Post Regression (n=47)	p-value* Baseline vs Post Treatment	p-value* Baseline vs Post Regression	Baseline (n=47)	Post Treatment (n=47)	Post Regression (n=47)	p-value* Baseline vs Post Treatment	p-value* Baseline vs Post Regression
Dryness:				<0.001	<0.001				<0.001	<0.001
None	1 (2.13%)	34 (72.3%)	45 (95.7%)			1 (2.13%)	33 (70.2%)	45 (95.7%)		
Mild	36 (76.6%)	13 (27.7%)	2 (4.26%)			35 (74.5%)	14 (29.8%)	2 (4.26%)		
Moderate	10 (21.3%)	0 (0.00%)	0 (0.00%)			11 (23.4%)	0 (0.00%)	0 (0.00%)		
Severe	0 (0.00%)	0 (0.00%)	0 (0.00%)			0 (0.00%)	0 (0.00%)	0 (0.00%)		
Scaling:				<0.001	<0.001				<0.001	<0.001
None	0 (0.00%)	9 (19.1%)	30 (63.8%)			0 (0.00%)	12 (25.5%)	32 (68.1%)		
Mild	28 (59.6%)	34 (72.3%)	15 (31.9%)			29 (61.7%)	34 (72.3%)	15 (31.9%)		
Moderate	16 (34.0%)	4 (8.51%)	2 (4.26%)			16 (34.0%)	1 (2.13%)	0 (0.00%)		
Severe	3 (6.38%)	0 (0.00%)	0 (0.00%)			2 (4.26%)	0 (0.00%)	0 (0.00%)		
Itching:				<0.001	<0.001				<0.001	<0.001
None	0 (0.00%)	12 (25.5%)	25 (53.2%)			0 (0.00%)	14 (29.8%)	24 (51.1%)		
Mild	11 (23.4%)	33 (70.2%)	22 (46.8%)			6 (12.8%)	29 (61.7%)	23 (48.9%)		
Moderate	26 (55.3%)	2 (4.26%)	0 (0.00%)			34 (72.3%)	4 (8.51%)	0 (0.00%)		
Severe	10 (21.3%)	0 (0.00%)	0 (0.00%)			7 (14.9%)	0 (0.00%)	0 (0.00%)		

Subjects' self-evaluation of dandruff symptoms, scalp and hair condition

**Figure 4:**
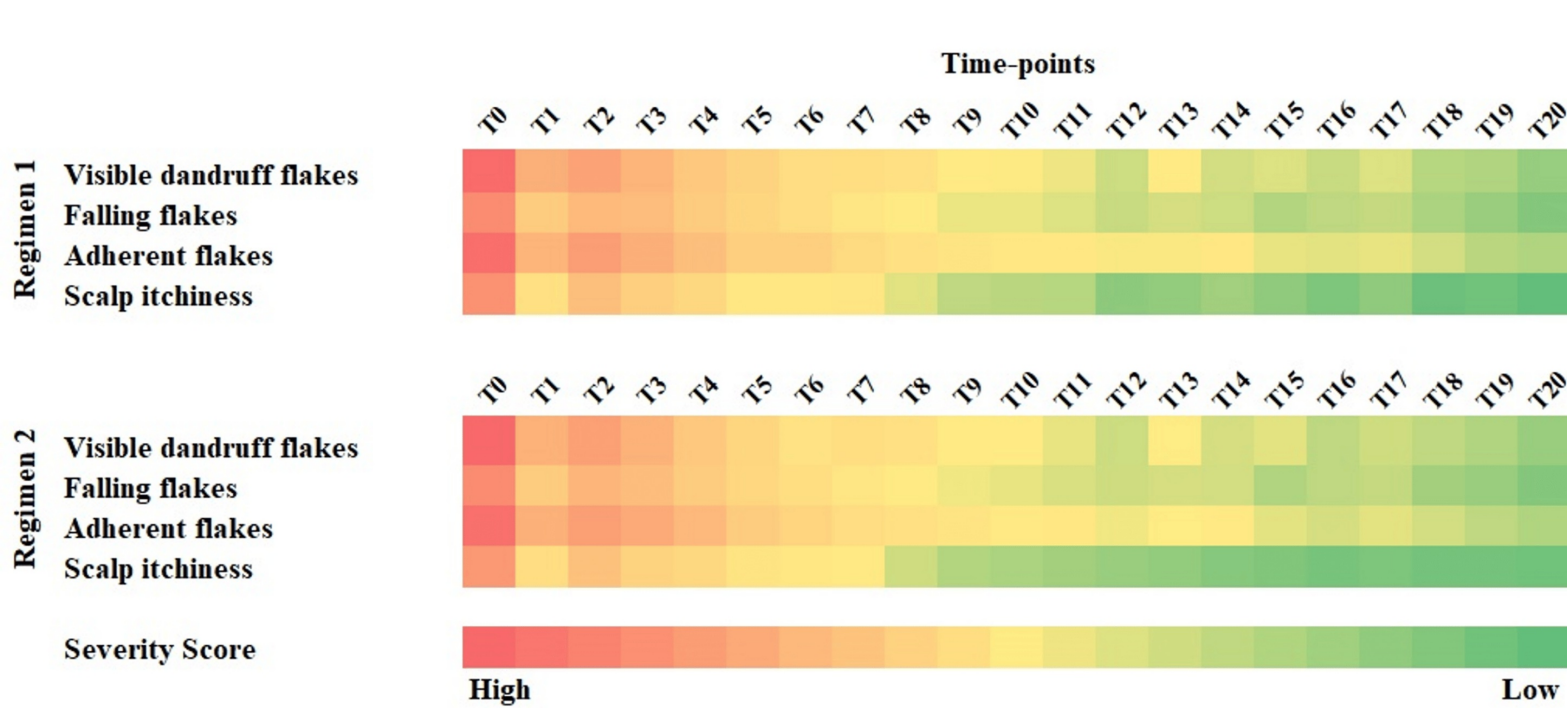
Subjects’ self-assessment of dandruff severity and scalp pruritis after using antiseptic liquid followed by use of non-anti-dandruff shampoo (Regimen 1) and placebo antiseptic liquid followed by use of anti-dandruff shampoo (Regimen 2), p-value >0.05, by t-test

**Figure 5:**
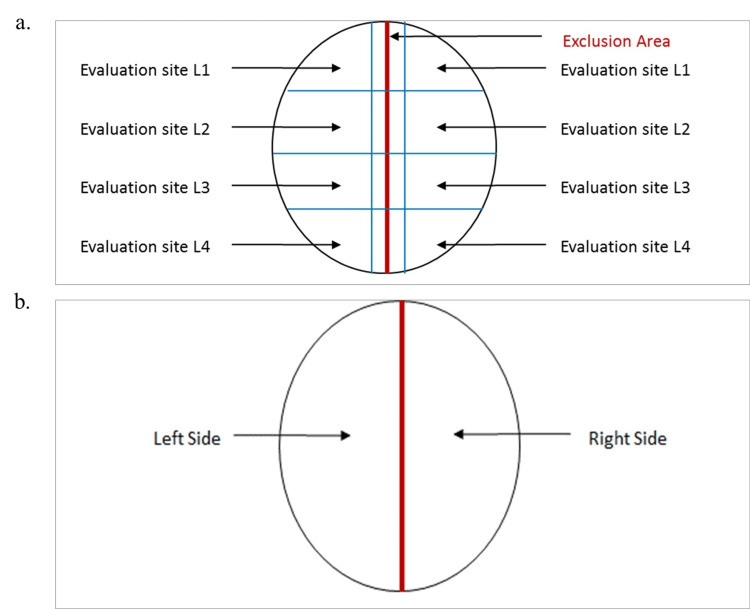
Illustration of scalp hair sectioning to a. aid assessment b. hair wash

The self-investigation of dandruff manifestation by the study population reported moderate itchiness and the visible, adherent, and falling flakes at baseline on either side of the scalp, and both the regimens were equally efficacious in conferring a significant and progressive reduction in scalp itchiness, visible, adherent, and falling flakes from the first wash itself and at all studied timepoints (p<0.001) (Figure 4). Both regimens conferred considerable improvement in scalp health and overall hair condition from the 1st wash itself and at all studied time points (p<0.001).

Dropouts/Discontinuation

One individual dropped out of the study due to personal reasons.

## Discussion

Chlorhexidine is widely used as an antiseptic and disinfectant agent. Its combination with cetrimide imparts synergistic effects on antimicrobial efficacy. In addition to its well-known broad-spectrum antibacterial activity, the chlorhexidine gluconate and cetrimide combination exhibited effective inhibitory action on various species of *Malassezia*, including *Malessezia furfur*, *M. globosa*, and *M. restricta*, which are implicated in dandruff. The presence of the *Malassezia* species, directly and indirectly, enhances the dandruff on the scalp, and most of the anti-dandruff formulations impart anti-dandruff activity by reducing the fungal load in the affected area [13]. In recent times, resistance against anti-fungal molecules has been a growing concern, and there is a need for newer molecules with alternate mechanisms of action [14].

The current treatment strategies to tackle *Malasssezia spp.* include anti-fungal drugs such as ketoconazole (KTZ), miconazole, zinc pyrithione, piroctone olamine, salicylic acid, selenium sulfide, and lipase inhibitors [15]. Out of these zinc pyrithione (ZnPTO), piroctone, olamine, and azole drugs are most commonly used to tackle seborrheic dermatitis. The azole drugs like ketoconazole and miconazole-based shampoos are the medicated formulations prescribed to treat severe dandruff conditions. Prolonged use of these anti-dandruff agents may lead to reduced effectiveness over time. Studies have found that prolonged use and exposure to azole drugs can result in the development of drug resistance in *Malassezia spp.* [16]. Also, these agents require a proper delivery vehicle for the proper availability of the site of action.

The present study was aimed at investigating the efficacy of a chlorhexidine+cetrimide antiseptic liquid on dandruff symptoms and related discomfort. Further, we intended to assess the efficacy of this combination in delaying the recurrence of dandruff compared to the reference product. The clinical study was conducted with a total number of 50 participants. Multiple studies have been conducted with a number of participants ranging from 25 to 50 to reach statistical conclusiveness. Godse et al. (2024) have shown the effectiveness of selenium sulfide in the treatment of dandruff with 30 subjects [17]. Also, significant results were obtained in a study on itch-relieving anti-dandruff shampoo formulation with only twenty participants [18].

This clinical trial was designed as a single-centered, double-blind, randomized, placebo-controlled, split-head paired design to study the tolerability and efficacy of the test products in conferring improved scalp health and hair attribute benefits in three phases. These phases included pre-conditioning of the scalp and hair, treatment with the test product, and finally, assessing the re-occurrence of the dandruff symptoms in the regression phase. Recently, it has been shown that this split-head paired design shows a similar ability to detect anti-dandruff test product efficacy as a whole-head parallel design [19]. The placebo liquid, followed by a commercially available zinc pyrithione-containing anti-dandruff shampoo, was used as a control in this study.

The chlorhexidine and cetrimide-based antiseptic liquid effectively alleviated dandruff and its symptoms compared to baseline (a mean reduction of ~71.89% and ~85.47% after the treatment phase and the regression phase, respectively) and performed at par with the commercially available shampoo containing zinc pyrithione. Both these active molecules work by inhibiting the dandruff-causing microorganisms. While the chlorhexidine and cetrimide-based antimicrobials exhibit antifungal activity by damaging the cell wall and the plasma membrane, the presence of ZnPTO leads to an increase in cellular zinc levels, inhibition of mitochondrial function, and a decrease in lipase expression in *Malassezia* fungal species [20].

The loose dandruff was assessed using the black card method, while the dermatologist assessed the scalp for itchiness and hair fall. The use of antiseptic liquid conferred a reduction of multiple symptoms associated with dandruff, including visible falling and adherent dandruff flakes, scalp itchiness, and hair fall. The decreased visible flakes and itchiness during the regression phase and at the end of the study can be attributed to a possible residual anti-microbial activity of both treatments. It could also be an indication of the effectiveness of the anti-microbial activity during the treatment phase.

The perception of study participants in terms of the reduction in dandruff and enhanced hair attributes is an important parameter to be considered while evaluating the efficacy of the product. The subjects assessed the efficacy of the product based on the questionnaire. The visible dandruff flakes were reduced after the treatment phase and remained reduced till the end of the study. The falling and adherent flakes were also reduced significantly (p<0.001) as assessed by the participants. The itchiness in the scalp observed initially was reduced to a significant level during treatment and the regression phase. Therefore, it can be said that apart from the dermatologists’ assessment, the personal experience of the participants was also significantly enhanced in terms of reduced dandruff symptoms. 

Although most antidandruff shampoos relieve the dandruff symptoms, they often leave hair in a condition that many consumers find unsatisfactory. In this situation, the propensity to return to the use of non-anti-dandruff shampoos increases, leading to a relapse of dandruff symptoms. This study reveals a potential solution to this compliance issue wherein it permits afflicted individuals to use this antiseptic liquid as a pre-treatment, followed by a shampoo of their choice.

No adverse event was reported in the present study that was related to the tested product. Some limitations of this study include the partially subjective nature of the assessments, recall bias, and the limited subject size. Nevertheless, we observed a statistically significant improvement in all subjects at a speed and extent comparable to the standard regimen. Therefore, the present study has proven the efficacy of a chlorhexidine-cetrimide-based anti-dandruff regimen to be on par with the ZnPTO regimen. The regimen comprising the Chlorhexidine-Cetrimide scalp rinse prior to a wash with non-anti-dandruff shampoo can impart similar effects to the dandruff symptoms as ZnPTO-based anti-dandruff shampoo. Considering the ease of use, efficacy, safety, lower economic value, and lower chances of the development of resistance, this strategy can be considered a newer regimen and an alternative to the current anti-dandruff strategies.

## Conclusions

The chlorhexidine-cetrimide combination is widely used as an antiseptic and disinfectant. We first established the *in vitro* efficacy of this combination against *Malassezia spp.* Given the *in vitro* efficacy and its proven safety for topical human use, we designed a clinical trial of this combination to test its efficacy against dandruff in comparison to a ZnPTO-containing anti-dandruff shampoo. This combination significantly reduced visible dandruff and related sensorial symptoms in all subjects, with a speed and extent at par with the standard regimen. It did not adversely affect the hair integrity and, in fact, reduced the hair fall count. These results establish that for individuals with moderate dandruff, the regimen of chlorhexidine and cetrimide-based antiseptic liquid for scalp treatment followed by a shampoo rinse can be as effective as a zinc-pyrithione-containing shampoo.

## Appendices

**Table 3:** Time points for the assessment of hair and scalp condition

Visit	Time point	Visit Description
1	T_0_, T_1_	Baseline, 1^st^ product application
2	T_2_	2^nd^ application
3	T_3_	3^rd^ application
4	T_4_	4^th^ application
5	T_5_	5^th^ application
6	T_6_	6^th^ application
7	T_7_	7^th ^application
8	T_8_	8^th^ Application commencement
9	T_9_	Regression Phase – 1^st^ Wash
10	T_10_	Regression Phase – 2^nd^ Wash
11	T_11_	Regression Phase – 3^rd^ Wash
12	T_12_	Regression Phase – 4^th^ Wash
13	T_13_	Regression Phase – 5^th^ Wash
14	T_14_	Regression Phase – 6^th^ Wash
15	T_15_	Regression Phase – 7^th^ Wash
16	T_16_	Regression Phase – 8^th^ Wash
17	T_17_	Regression Phase – 9^th^ Wash
18	T_18_	Regression Phase – 10^th^ Wash
19	T_19_	Regression Phase – 11^th^ Wash
20	T_20_	Regression Phase – 12^th^ Wash and study completion

**Table 4:** Scoring matrix for dandruff severity assessment by the dermatologist

Score	Area Affected	Flake Type and Dandruff severity
0	0%	No flakes
1	1-20%	Small powdery flakes, lightly covering the scalp
2	21-40%	Slightly larger flakes loosely present on the scalp
3	41-60%	Large flakes loosely attached to scalp giving an irregular whitish surface
4	61-80%	Large Flakes adherent to scalp slightly firmly
5	81-100%	Flakes apparently congealed together into yellowish plates adhering to scalp, sometimes with evidence of exudate and erythema

**Table 5:** Scalp itching severity scale

Score	Itch severity
1	No itching
2	Mild itching (Occasional)
3	Moderate itching (frequent itching, at least 5-8 times in a day)
4	Severe itching (more frequent itching disturbing daily routine)
5	Very severe itching (disturbing night sleep)

**Table 6:** Area covered by loose dandruff flakes

Score	Area Covered by Loose flakes
1	0% coverage
2	1-20% coverage
3	21 to 40% coverage
4	41 to 60% coverage
5	61 to 80% coverage
6	81 to 100% coverage

**Table 7:** Scoring matrix for subject’s self-assessment of scalp and hair condition

Scalp condition	Score 1	Score 10
Itching	None	Severe
Oiliness	Non-oily	Very oily
Dryness	Not dry	Very dry
Visible dandruff	None	Numerous
Falling flakes	None	Numerous
Adhering flakes	None	Numerous
Scalp health	Poor	Very healthy
Hair condition	Score 1	Score 10
Softness	Not soft	Very soft
Smoothness	Very rough	Very smooth
Moisturized feel	Very dry	Very moisturized
Combability	Very difficult	Very easy
Static	Very high static	Very low static
Manageability	Very difficult	Very easy
Ease of detangling	Very difficult	Very easy
Shine	Very dull	Very shiny
Silkiness	Non-silky	Very silky
Stickiness	Very sticky	Non-sticky
Hair breakage/fall	Very high	Very low
Overall hair condition	Poor	Excellent

**Table 8:** Scoring matrix for safety evaluation of product based on the dermatologist assessment

Score	Description		Clinical Signs	Functional signs
1	None		Erythema	Itching
2	Mild		Oedema	Tingling
3	Moderate		Dryness	Stinging
4	Severe		Scaling	Burning
5	Very severe		Peeling	Other

## References

[1] Ranganathan S, Mukhopadhyay T (2010). Dandruff: the most commercially exploited skin disease. Indian J Dermatol.

[2] Rudramurthy SM, Honnavar P, Dogra S, Yegneswaran PP, Handa S, Chakrabarti A (2014). Association of Malassezia species with dandruff. Indian Journal of Medical Research.

[3] Gaitanis G, Magiatis P, Hantschke M, Bassukas ID, Velegraki A (2012). The Malassezia genus in skin and systemic diseases. Clin Microbiol Rev.

[4] Park M, Park S, Jung WH (2021). kin commensal fungus Malassezia and its lipases. J Microbiol Biotechnol.

[5] Dawson TL Jr (2007). Malassezia globosa and restricta: breakthrough understanding of the etiology and treatment of dandruff and seborrheic dermatitis through whole-genome analysis. J Investig Dermatol Symp Proc.

[6] Turner GA, Hoptroff M, Harding CR (2012). Stratum corneum dysfunction in dandruff. Int J Cosmet Sci.

[7] Ruff ML, McClanahan SB, Babel BS (2006). In vitro antifungal efficacy of four irrigants as a final rinse. J Endod.

[8] Shailaja S, Bhat SS, Hegde SK (2013). Comparison between the antibacterial efficacies of three root canal irrigating solutions: antibiotic containing irrigant, Chlorhexidine and Chlorhexidine + Cetrimide. Oral Health Dent Manag.

[9] (2024). Guideline IH. E6: Note for guidance on good clinical practice. https://www.ema.europa.eu/en/ich-e6-r2-good-clinical-practice-scientific-guideline.

[10] Bošnjak S (2001). The declaration of Helsinki: The cornerstone of research ethics. Arch Onc.

[11] Mathur R, Swaminathan S (2018). National ethical guidelines for biomedical &amp; health research involving human participants, 2017: A commentary. Indian J Med Res.

[12] Bacon RA, Mizoguchi H, Schwartz JR (2014). Assessing therapeutic effectiveness of scalp treatments for dandruff and seborrheic dermatitis, part 1: a reliable and relevant method based on the adherent scalp flaking score (ASFS). J Dermatolog Treat.

[13] Shuster S (1984). The aetiology of dandruff and the mode of action of therapeutic agents. Br J Dermatol.

[14] Leong C, Kit JC, Lee SM, Lam YI, Goh JP, Ianiri G, Dawson TL Jr (2021). Azole resistance mechanisms in pathogenic M. furfur. Antimicrob Agents Chemother.

[15] Angiolella L, Carradori S, Maccallini C, Giusiano G, Supuran CT (2017). Targeting Malassezia species for novel synthetic and natural antidandruff agents. Curr Med Chem.

[16] Park M, Cho YJ, Lee YW, Jung WH (2020). Genomic multiplication and drug efflux influence ketoconazole resistance in Malassezia restricta. Front Cell Infect Microbiol.

[17] Godse G, Godse K (2024). Safety, efficacy and attributes of 2.5% selenium sulfide shampoo in the treatment of dandruff: a single-center study. Cureus.

[18] Lim DZ, Lim FC, Tey HL (2023). Clinical efficacy of a gentle anti-dandruff itch-relieving shampoo formulation. Int J Cosmet Sci.

[19] Diao Y, Matheson JR, Pi Y, Baines FL, Zhang S, Li Y (2021). Comparison of whole-head and split-head design for the clinical evaluation of anti-dandruff shampoo efficacy. Int J Cosmet Sci.

[20] Park M, Cho YJ, Lee YW, Jung WH (2018). Understanding the mechanism of action of the anti-dandruff agent zinc pyrithione against Malassezia restricta. Sci Rep.

